# Oxytocin modulates GABA_A_R subunits to confer neuroprotection in stroke *in vitro*

**DOI:** 10.1038/srep35659

**Published:** 2016-10-21

**Authors:** Yuji Kaneko, Colleen Pappas, Naoki Tajiri, Cesar V. Borlongan

**Affiliations:** 1 Center of Excellence for Aging and Brain, Department of Neurosurgery and Brain Repair, University of South Florida College of Medicine, 12901 Bruce B Downs Blvd, Tampa FL 33612, USA; 2 School of Aging Studies, University of South Florida, 13301 Bruce B Downs Blvd, Tampa FL 33612, USA

## Abstract

Oxytocin protects against ischemia-induced inflammation and oxidative stress, and is associated with GABA (γ-aminobutyric acid, an inhibitory neurotransmitter) signaling transduction in neurons. However, the molecular mechanism by which oxytocin affords neuroprotection, especially the interaction between oxytocin receptor and GABA_A_ receptor (GABA_A_R), remains to be elucidated. Primary rat neural cells were exposed to oxytocin before induction of experimental acute stroke model via oxygen-glucose deprivation-reperfusion (OGD/R) injury. Pretreatment with oxytocin increased cell viability, decreased the cell damage against oxidative stress, and prevented the release of high mobility group box1 during OGD/R. However, introduction of oxytocin during OGD/R did not induce neuroprotection. Although oxytocin did not affect the glutathione-related cellular metabolism before OGD, oxytocin modulated the expression levels of GABA_A_R subunits, which function to remove excessive neuronal excitability via chloride ion influx. Oxytocin-pretreated cells significantly increased the chloride ion influx in response to GABA and THIP (δ-GABA_A_R specific agonist). This study provides evidence that oxytocin regulated GABA_A_R subunits in affording neuroprotection against OGD/R injury.

Despite advances in the management and care of stroke, ischemic-reperfusion injury is still a major cause of mortality and morbidity. Male stroke incidence rate and prevalence are significantly higher than females worldwide^1^, indicating that this gender difference may be the result of sex hormone, i.e., estrogen. Estrogen regulates oxytocin synthesis in many organs, including the brain. Oxytocin is a typical stress hormone that responds to several acute and chronic stressors, and, together with its receptors, modulates an important array of physiological and biological activities^2^ in central and peripheral nervous systems, such as facilitating birth^3^. Estrogen, interleukin (IL)-1β, IL-6, interferon τ, and oxytocin regulate the expression levels of oxytocin receptors^4^.

Plasma oxytocin increases the expression of the peroxisome proliferator-activated receptor gamma gene, a regulator of adipocyte differentiation, and regulates the activity of eukaryotic elongation factor 2^5^, a translation-related protein. Oxytocin controls the differentiation of bone marrow-derived mesenchymal stem cells, regulates the cells’ proliferation and carbohydrate metabolism^6^, and promotes lipid metabolism as an energy substrate. Mice deficient in oxytocin receptors have been found to develop obesity^7^.

GABA is the principal inhibitory neurotransmitter in the brain and binds three receptors, GABA_A_R, GABA_B_R, and GABA_C_R. GABA_A_R plays a major role in fast synaptic inhibition in the central nervous system (CNS), and is activated by allosteric modulation of interfacial five compositions (2α/2β/1γ, δ, ε, θ, π) assembled from 16 subunits (α_1–6_, β_1–3_, γ_1–3_, δ, ε, θ, and π) consisting of 20 subtypes^8^. The GABA_A_R-mediated hyperpolarization of membrane potential is attributed to the direct activation of an integral anion channel, and the resultant influx of chloride ions along its electrochemical gradient^9^. The equilibrium shift of GABA_A_R subtype expression pattern is a key control point for the determination of receptor diversity of the neuronal plasma membrane. Appropriate equilibrium of inhibitory and excitatory neurotransmission regulates the neuronal network in normal brain function. Conversely, an imbalance between inhibitory and excitatory neurotransmission after an ischemic insult creates an excessive secretion of excitatory molecules and suppresses the GABAergic inhibition system, by selectively limiting the trafficking of GABA_A_Rs on the plasma membrane^10^.

Excitotoxicity has been well-documented as a causative factor in ischemia-induced neuronal cell death^11^. GABA_A_Rs on neuronal cell membrane are decreased when exposed to oxygen-glucose deprivation (OGD), suggesting that the number of membrane-bound GABA_A_Rs could be a pivotal process in the progression of ischemic-induced neuronal cell death^12^. Oxytocin regulates GABA_A_R-mediated synaptic signaling in the fetal brain during delivery, and reduces brain vulnerability to hypoxic damage^13^. Although oxytocin-induced neuroprotection has been demonstrated in ischemic-reperfusion injury models, the molecular mechanisms underlying such therapeutic benefit, especially how oxytocin interacts with individual GABA_A_R subtypes^14^, are still unknown.

In this study, we demonstrated that administration of oxytocin in primary rat neural cells (PRNCs) before OGD resulted in robust neuroprotective effects, but not when oxytocin was initiated during OGD/R. We also showed that oxytocin shifted the expression patterns of GABA_A_R subunit on the cells, accompanied by increased chloride ion influx. These observations provide evidence that oxytocin modulated GABA_A_R in exerting its neuroprotective effects against ischemia-induced neuronal cell death.

## Results

### Oxytocin exerts neuroprotection against OGD/R

**Figure 1:**
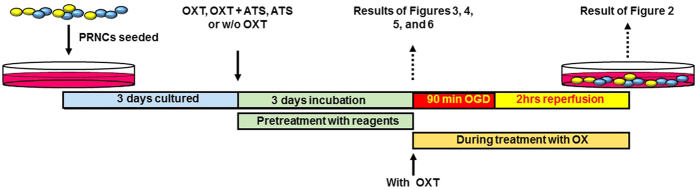
Experimental design. PRNCs; primary rat neural cells. OGD; oxygen and glycose deprivation. OXT; oxytocin. ATS; atosiban.

**Figure 2:**
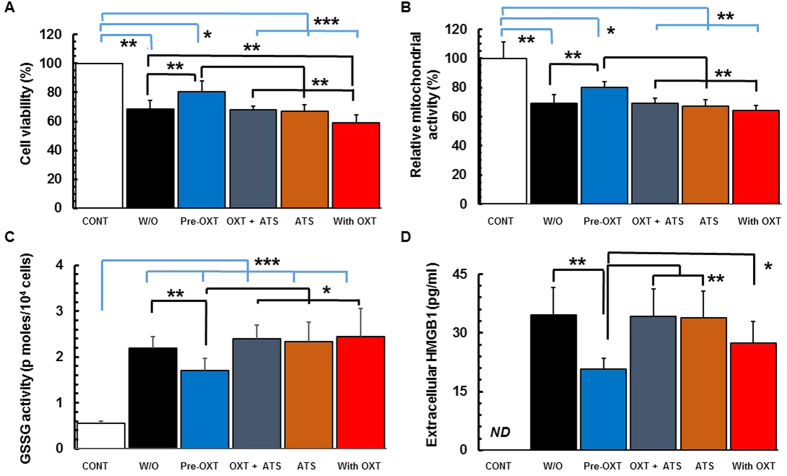
Oxytocin confers neuroprotective effects and attenuates the oxidative stress against OGD. (**A**) Cell viability tested by Calcine-AM/EthD-1 florescence and trypan blue dyes. (**B**) Mitochondrial activity by MTT assay. (**C**) GSSG activity. (**D**) Extracellular HMGB1 levels. *P < 0.05, **P < 0.01, and ***P < 0.001. Experiments were conducted in triplicate, with n = 6 per treatment condition in each run.

As shown in Fig. 1, PRNCs were exposed to the OGD/R *in vitro* model of stroke^15^,^16^. Pretreatment with oxytocin increased cell viability (*F*_(2,34)_ = 19.48; *P* < 0.0001; Fig. 2A), decreased mitochondrial damage (*F*_(2,25)_ = 31.81, *P* < 0.0001; Fig. 2B), reduced oxidative stress (*F*_(2,30)_ = 406, *P* < 0.0001; Fig. 2C), and prevented cell secretion of high mobility group box 1 (HMGB1), a mediator of ischemic progression^11^,^17^. Atosiban, the selective oxytocin receptor antagonist^13^, abolished these oxytocin receptor-mediated beneficial effects. Oxytocin administered during OGD/R had no neuroprotective effect (indicated as “With OXT” in Fig. 2).

### Biological activity readouts across treatments

**Figure 3:**
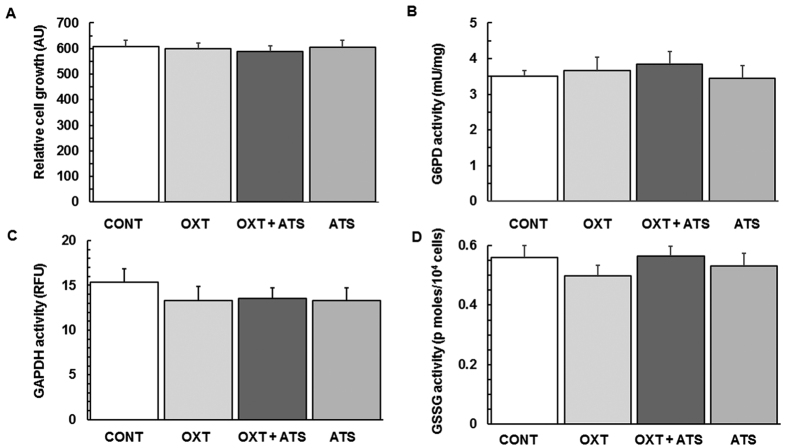
Effects of oxytocin treatment on biological activity before OGD. (**A**) PRNCs’ cell growth. Oxytocin had no significant effects on the cell growth, although oxytocin has been shown to contribute to the differentiation of bone marrow-derived mesenchymal stem cells^6^ (**B**) G6PD activity. G6PD catalyzes the rate-determining step in the pentose phosphate pathway and produces NADPH to regulate GSH/GSSG levels. G6PD activities across treatments were not significantly different. (**C**) GAPDH activity. GAPDH regulates the ATP generation phase of glycolysis-derived NAD and functions as a reversible metabolic switch under oxidative stress. GAPDH activities across treatments did not significantly differ. (**D**) GSSG activity. GSSG is a biomarker of oxidative stress, and is generated by oxidized GSH with reduced NADPH. There were no statistical differences across conditions. Experiments were conducted in triplicate, with n = 6 per treatment condition in each run.

**Figure 4:**
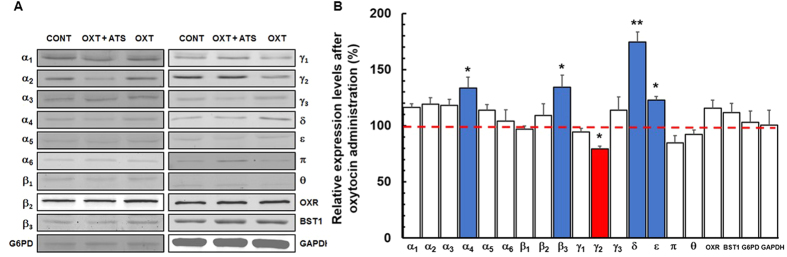
Western blot analysis. Following incubation of PRNCs in the absence of reagents (control; CONT), or with 1 μM oxytocin + 10 μM atosiban (OXT + ATS), or 1 μM oxytocin (OXT) for 3 days 37 °C. (**A**) Expression levels of GABA_A_R subunits, oxytocin receptor (OXR), bestrophin-1 (BST1), G6PD, and GAPDH. (**B**) Relative quantification of protein expression levels. Blue bars represent significantly increased protein expression levels, red bar showed significantly decreased levels, and white bars indicate no statistical differences between oxytocin-treated cells and control. *P < 0.05 and **P < 0.01. The dotted red line represents combined data from control and OXT + ATS, since these two groups did not significantly differ. Experiments were independently conducted in 3~6 times.

Oxytocin acts as an anabolic hormone, and exhibits cell growth^5^,^6^ and anti-oxidative properties, suggesting its potential therapeutic application in stroke^18^. However, oxytocin administration did not alter cell growth of PRNCs compared with control treatment (Fig. 3A). Next, because peripheral oxytocin participates in glucose metabolism in modulating reactive oxygen species (ROS) production via NADPH (nicotinamide adenine dinucleotide phosphate) pathway, we examined the effects of oxytocin on glutathione (a major antioxidant), and glucose 6-phosphate dehydrogenase (G6PD) and glyceraldehyde 3-phosphate dehydrogenase (GAPDH) (key enzymes that regulate NADPH production)^19^. Results revealed that G6PD, GAPDH, and glutathione disulfide (GSSG) activity levels did not significantly differ across treatment conditions (Fig. 3B–D). Pretreating the cells with oxytocin did not change the protein expression levels of G6PD and GAPDH in comparison with control values (Fig. 4).

### Oxytocin pretreatment shifts the GABA_A_R subunit expression patterns

Oxytocin has been found to alter the subtype expression patterns^20^ and function of GABA_A_Rs^13^,^21^. We therefore assessed whether oxytocin modulated the expression patterns of GABA_A_Rs subunit on PRNCs (Fig. 4). Treatment of PRNCs with oxytocin significantly increased α_4_, β_3_, δ, and ε GABA_A_R subunit expression levels, but decreased γ_2_ GABA_A_R subunit (Fig. 4). Although oxytocin receptor expression has been reported to be increased following oxytocin treatment^4^, we could not detect any differences in oxytocin receptor upregulation between control and oxytocin treatment in this experiment (Fig. 4). Moreover, oxytocin treatment did not affect the expression levels of Bestrophin-1 (BST1), a calcium-activated chloride ion channel normally distributed on synapses adjacent to soma^22^ and shown to mediate GABA release from astrocytes^23^.

### Localization of GABA_A_R subunits

**Figure 5:**
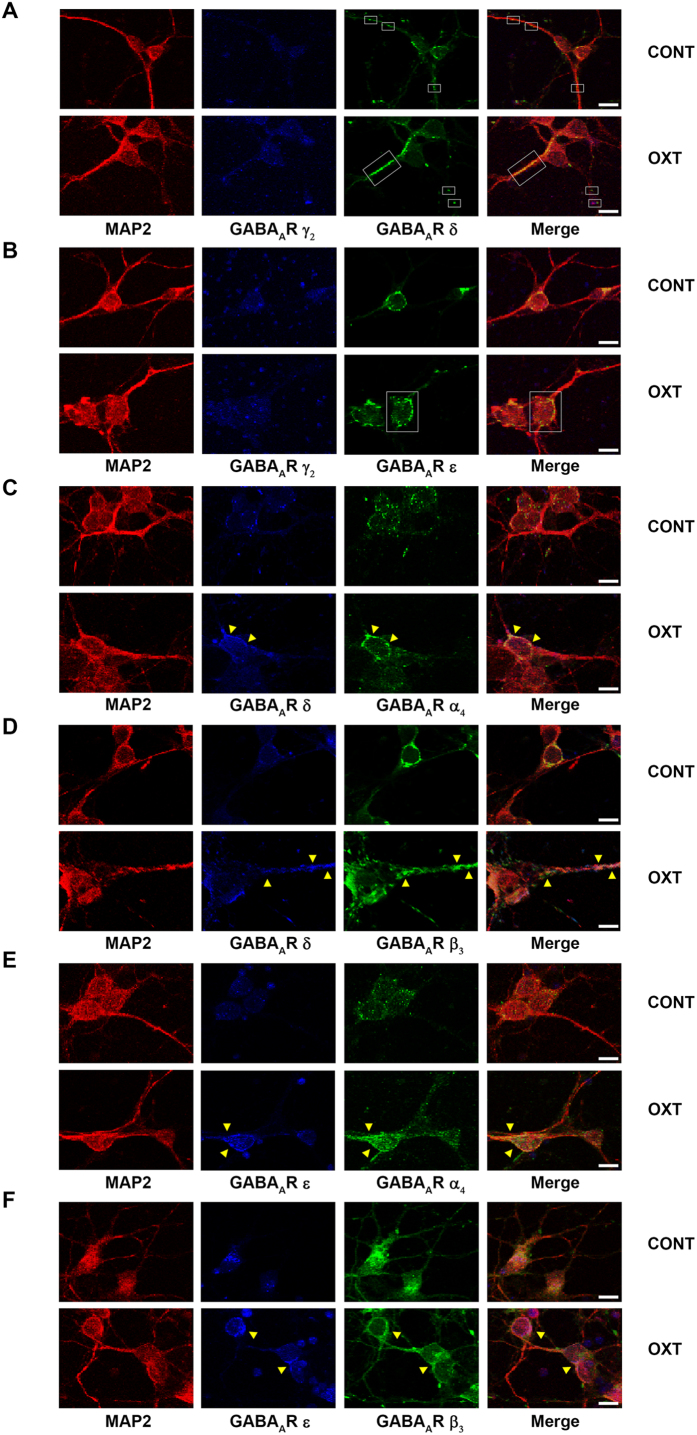
Localization of δ, γ_2_, α_4_, β_3_, and ε GABA_A_R subunits expression on neurons. Following incubation of PRNCs in the absence of reagent (control; CONT) or with 1 μM oxytocin (OXT) for 3 days 37 °C. (**A**) Expression of γ_2_ and δ-subunits, (**B**) γ_2_ and ε-subunits, (**C**) δ and α_4_-subunits, (**D**) δ and β_3_-subunits, (**E**) ε and α_4_-subunits, and (**F**) ε and β_3_-subunits. Scale bars = 10 μm.

Electric current and localization patterns of GABA_A_R vary depending on the region of interest within the neuron, because glycogen (the main storage form of glucose in the body) is predominantly preserved in the soma where the main production of ATP occurs. In the ischemic brain, the rate of glycogen metabolism is significantly increased^24^. We observed the localization of α_4_, β_3_, γ_2_, δ, and ε GABA_A_R subunit expression within subcellular compartments of the neuron. Immunocytochemichal analysis showed that (i) δ GABA_A_R subunit was mainly located at the axon (Fig. 5A, indicated with box), (ii) ε GABA_A_R subunit predominantly resided in the soma (Fig. 5B, indicated with box), (iii) γ_2_, α_4_, and β_3_ GABA_A_R subunits were broadly expressed in the whole neuron (Fig. 5A,B,E,F), (iv) α_4_/δ- and β_3_/δ-GABA_A_R subunits showed co-localization (Fig. 5C,D, indicated with arrow), and (v) α_4_/ε- and β_3_/ε-GABA_A_R subunits were also co-localized in PRNCs (Fig. 5E,F, indicated with arrow).

### Intracellular chloride ion influx kinetics

**Figure 6:**
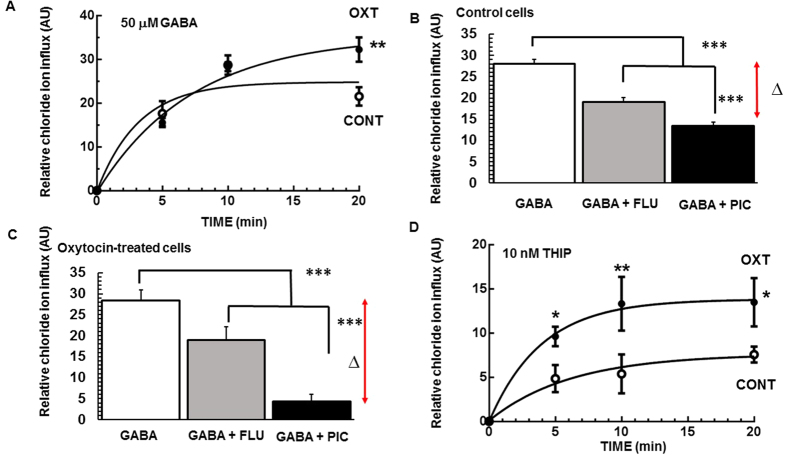
Characterization of chloride ion influx via GABA_A_R. Following incubation of PRNCs in the absence of reagents (control; CONT) or with 1 μM oxytocin (OXT) for 3 days 37 °C. (**A**) Time course of chloride ion influx stimulated by 50 μM GABA. (**B**,**C**) The cells were pretreated with 1 μM flumazenil (GABA + FLU), or 1 μM picrotoxin (GABA + PIC), or PBS (GABA) for 45 min at 37 °C, and were stimulated by 50 μM GABA for 10 min at RT, and then fluorescence intensity was measured. (**B**,**C**) represent control- and oxytocin treated-cells, respectively. The Δ value was calculated from following equation: fluorescence intensity of (GABA – [GABA + PIC]). (**D**) Time course of chloride ion influx stimulated by 10 nM THIP. *P < 0.05, **P < 0.01, and ***P < 0.001. Experiments were conducted in triplicate, with n = 6 per treatment condition in each run.

After binding with GABA, GABA_A_R engages a chloride ion selective pore, resulting in chloride ion influx that inhibits the firing of neuron action potentials. The kinetic property of GABA_A_R depends on receptor subunit compositions, thereby providing a mechanism for neurons to regulate individual biological activities. We performed a time course study to reveal any differences in GABA-elicited chloride ion influxes between control- and oxytocin-treated PRNCs. Figure 6A revealed that chloride ion influx reached equilibrium at 10 min, but treatment with oxytocin significantly increased the influx at the 20 min period. To assess the differences of both GABA_A_R antagonistic conditions, we compared the inhibition dynamics of GABA-induced chloride ion influx in the presence of flumazenil (GABA_A_R antagonist, GABA + FLU) or picrotoxin (GABA_A_R channel blocker, GABA + PIC). Both reagents inhibited the GABA-induced chloride ion influx (control; Fig. 6B and oxytocin treatment; Fig. 6C). Interestingly, oxytocin-treated cells were more sensitive to picrotoxin inhibition, as evidenced by the Δ value of control cells = 14.0 ± 2.20 AU (Fig. 6B), and that of oxytocin-treated cells = 24.1 ± 1.70 AU (Fig. 6C), *P* < 0.001. Because δ GABA_A_Rs display increased sensitivity to THIP (δ-GABA_A_R specific agonist)^25^, we tested whether oxytocin-treated cells additively increased THIP-induced chloride ion influx. Results revealed that THIP-evoked chloride ion influx of oxytocin-treated cells was significantly higher than that of control (Fig. 6D).

## Discussion

The present study revealed a novel molecular mechanism underlying oxytocin-mediated neuroprotection against ischemic stroke in a cell culture paradigm. We found that oxytocin-induced GABA_A_R subunit modification is a predominant factor in conferring neuroprotection against OGD. GABA is the principal inhibitory transmitter in the brain, and its functions are mediated by ubiquitously expressed ligand-opened chloride ion channel GABA_A_Rs^26^. Aberrant GABAergic inhibition is a key pathological feature displayed by ischemic neurons in the peri-infarct area (secondary damaged region) after stroke^26^. Our present results demonstrated that oxytocin reduced ischemic stroke deficits likely by modulating specific GABA_A_R subtype signal transduction^14^, which parallels studies showing that oxytocin improves stroke outcomes via social interaction pathways^18^.

We showed that oxytocin protected PRNCs against OGD (Fig. 2). Ischemic injury is mediated by ROS, generated primarily by damaged mitochondria^27^, which leads to apoptosis and necrosis. During OGD, cell viability and mitochondrial activity were decreased, and the GSSG activity and extracellular HMGB1 levels were increased. HMGB1, a non-histone DNA-binding protein, is released from necrotic neurons after 2 h OGD^17^, and its concentrations in serum are significantly increased in stroke patients due to blood brain barrier (BBB) disruption associated with the disease progression^11^,^17^. That oxytocin exerted neuroprotection in OGD, but not in the OGD/R model is consistent with *in vivo* evidence, demonstrating that the subsequent reperfusion after ischemia exacerbates neuronal functions and causes massive brain injuries when oxygen-saturated and nutrient-rich blood suddenly returns to the lesion after a period of ischemia^11^, suggesting that OGD/R is worse than OGD. Under the OGD condition, pretreatment with oxytocin increased cell viability and mitochondrial activity, decreased the GSSG activity, and prevented HMGB1 secretion from the cells. In the presence of atosiban, this neuroprotection was abolished, indicating that the therapeutic effect was likely mediated by oxytocin receptor signal transduction. HMGB1 is phosphorylated by protein kinase C^28^ and calcium/calmodulin-dependent protein kinase^29^. Although oxytocin is capable of activating both kinases^3^, we could not detect extracellular HMGB1 despite incubating the cells with oxytocin prior OGD. Altogether, these observations suggest that oxytocin could serve as a neuroprotective agent in the acute phase of stroke by acting as an ischemic preconditioning factor in modulating therapeutic protein synthesis.

Oxytocin regulates glucose uptake that is critical for stem cell growth^6^ and antioxidant activity^30^. However, cell growth of PRNCs was not affected by oxytocin (Fig. 3A). We thus tested whether oxytocin utilized its receptor signal transduction in regulating glutathione-related proteins (G6PD, GAPDH, GSSG). G6PD regulates the antioxidant activity of NADPH^31^, facilitating NADPH to maintain glutathione/GSSG recycling^32^. GAPDH is not only a key enzyme in glycolysis, but also phosphorylates the α_1_ GABA_A_R subunit for sustaining the GABA_A_R structure and stability, thereby establishing the link of GABAergic inhibition with glucose metabolism. Under ischemic condition, glycolytic flux increases, while GAPDH activity is reversibly decreased, inhibiting neuronal cells from producing NADPH^31^ as a result of increased G6PD activity. We speculated that oxytocin would normalize G6PD, GAPDH, GSSG activity, but no significant difference were detected across treatment conditions (Fig. 3B–D), suggesting that oxytocin’s neuroprotection is largely independent of G6PD, GAPDH, and GSSG signal transduction pathways.

Maternal oxytocin exerts neuroprotective action on fetal neurons during parturition (a perturbed physiological environment similar to hypoxic-ischemic brain) mediated by GABA_A_R signaling pathway^13^. In the present study, oxytocin likely engaged the GABA_A_R subunit expression patterns by enhancing α_4_, β_3_, δ, and ε GABA_A_R subunit expression levels while reducing γ_2_ GABA_A_R subunit on PRNCs (Fig. 4). Although most GABA_A_R subunit expressions were not significantly influenced in the presence of atosiban (OXT + ATS), α_2_ GABA_A_R subunit expression decreased and γ_1_ and π GABA_A_R subunits increased (Fig. 4A), implicating that redundant signal impedance of oxytocin receptor could activate alternative signal transduction of these subunit expressions. Conversely, an on/off signaling switch of oxytocin may be tightly regulated by engagement with GABA_A_R subunits.

The β_3_ and γ_2_ GABA_A_R subunits on the neuronal membrane are vulnerable to ischemic stroke^10^,^33^. In the late stage of rat pregnancy the maternal brain displays increased expression of the ε GABA_A_R subunit, which is responsible for the respiratory function^34^. The α_4_ GABA_A_R subunit, on the other hand, is associated with dendritic development^35^, and is well co-expressed with δ-subunit in the brain^8^,^36^. The α_4_, δ, and ε GABA_A_R subtypes are extrasynaptic GABA_A_Rs^8^,^36^, which mediate tonic inhibition upon activation by GABA spillover from synaptic sites, as well as by ambient GABA in the extracellular space. The majority of GABA_A_Rs are α_1_β_2_γ_2_, α_2_β_3_γ_2_, and α_3_β_2/3_γ_2_, approximately occupying 80% of total GABA_A_R expressions in the brain^8^, in contrast, α_4_, δ, and ε GABA_A_Rs represent less than 5%^8^. Oxytocin-treated cells significantly increased α_4_, δ, and ε GABA_A_R subunit expression levels and decreased γ_2_ GABA_A_R subunit on PRNCs (Fig. 4). It is conceivable that oxytocin pretreatment led to an upregulation of specific GABA_A_R subtype expression levels, which in turn might have modified the neuronal networks towards neuroprotection.

Electrical properties of a neuron vary along the segments of subcellular organization (soma, dendrites, and axon), which is essential for orchestrating cellular function and structure preservation^37^. GABA_A_R-mediated chloride ion fluctuation substantially changes the intracellular chloride ion concentration in the soma and spreads into the dendrites^38^. Hypoxic ischemia causes retrograde neurodegeneration, which shortens the axonal and dendritic lengths and swells the soma, and produces a rapid and significant loss of axon in the acute phase of injury^39^. Therefore, it is an important to elucidate the GABA_A_R subunit localizations at the subcellular neuron. Interestingly, δ GABA_A_R subunit is mainly expressed at axon, and ε GABA_A_R subunit is primarily distributed on soma which concurred with previous report^40^. In contrast, α_4_, β_3_ and γ_2_ GABA_A_R subunits were broadly expressed throughout the whole neuron^8^. Here, we found that α_4_/δ- and β_3_/δ-GABA_A_R subunits, and α_4_/ε- and β_3_/ε-GABA_A_R subunits were well co-localized on neurons (Fig. 5). That appropriate distribution and specific expression of GABA_A_Rs subtypes exist in neurons suggest that oxytocin could elicit neuroprotection by subcellularly targeting specific GABA_A_R subunits within ischemic neurons.

Under hypoxic ischemia, the extracellular GABA concentration on/around the synaptic cleft increases and elevates neuronal intracellular chloride ion, which functions as a counter-reaction of depolarization^41^. We demonstrated here that oxytocin modulated discrete GABA_A_R subunits tasked to monitor chloride ion influx. The kinetic of GABA-stimulated chloride ion influx on oxytocin-treated cells was altered (Fig. 6A), in that while the response to GABA by the control cells was saturated at 10 min, oxytocin-treated cells continuously evoked the ion influx for 20 minutes, suggesting that boosting GABA_A_Rs-mediated neuronal inhibition can afford substantial protection while minimizing the extent of neuronal cell loss during OGD. Following incubation of cells with 50 μM GABA for 10 min (Fig. 6A), the values of chloride ion influx were similar. We next assessed GABA_A_R antagonism and found that Flumazenil inhibited the γ contained GABA_A_Rs in response to GABA, but was not able to antagonize the δ and ε GABA_A_Rs^42^. Picrotoxin directly binds the ion pore of GABA_A_R, thereby regulating the influx of chloride ion, and inhibiting the whole GABA_A_R channel activity^42^. The inhibition ability of flumazenil (GABA + FLU) did not significantly differ between conditions, implicating that oxytocin administration had no effect on the expression levels of γ GABA_A_R subtypes (Fig. 6B,C). In contrast, oxytocin-treated cells exhibited significant inhibition of chloride ion influx following picrotoxin treatment (GABA + PIC) compared to control, suggesting that oxytocin significantly increased total GABA_A_R expression, especially δ and ε GABA_A_R subtypes. Of note, to date, there is no specific antagonist for δ and ε GABA_A_R subunits. The present observation of specialized GABA_A_Rs antagonism is also supported by δ-GABA_A_R specific agonist THIP significantly elevating the chloride ion influx of oxytocin-treated cells compared to control (Fig. 6D). In summary, oxytocin induced the shift of GABA_A_R subunit expression in cultured PRNCs, which likely changed the kinetics of chloride ion influx in response to GABA.

We demonstrated that oxytocin exerts neuroprotection against ischemic stroke, but requires its treatment initiation prior to injury induction. Oxytocin may serve as a pharmacological ischemic preconditioning factor that can engage GABA_A_R towards neuroprotection. The present results provide evidence that oxytocin altered the expression patterns of GABA_A_R subunit and the kinetics of GABA-induced chloride ion influx. Our study highlights a close interaction between oxytocin and GABA_A_R that should aid in our understanding of stroke pathology and its treatment.

## Methods

### Cell culture and oxygen-glucose deprivation-reperfusion (OGD/R) progression

Primary rat neural cells (PRNCs; consisted of 40% neurons and 60% astrocytes) were obtained from BrainBit (E18 rat cortex; Springfield, IL, USA). As described elsewhere^15^, cells (4 × 10^4^ cells/well) were suspended in 200 μl Neural Medium (NbActive 4, BrainBit) containing 2 mM *l*- glutamine and 2% B27 in the absence of antibiotics and grown in poly-*l*-lysine-coated 96-well plates at 37 °C in humidified atmosphere containing 95% O_2_ and 5% CO_2_. After 3 days in culture, PRNCs were exposed to 1 μM oxytocin (O4375, Sigma-Aldrich, St. Louis, MI, USA), 1 μM oxytocin + 10 μM atosiban (A3480, Sigma-Aldrich), 10 μM atosiban, and the absence of regents (control) for 3 days at 37 °C. After 6 days in culture (Fig. 1), RPNCs were exposed to OGD as described previously^15^. The cells were initially exposed to OGD medium (glucose-free Dulbecco’s Modified Eagle Medium), then placed in an anaerobic chamber containing 95% N_2_ and 5% CO_2_ for 15 min at 37 °C (preincubation), for 90 min at 37 °C (culture medium pH 6.7~6.8; mimicking the acidic environment of ischemic brain *in vivo*). OGD was terminated by adding 5 mM glucose to medium and cell cultures were re-introduced to the regular CO_2_ incubator at 37 °C for 2 h. Control cells were incubated in the same buffer containing 5 mM glucose at 37 °C in a regular 95% O_2_ and 5% CO_2_ incubator.

### Measurement of cell viability

Measurement of cell viability was performed using fluorescent live/dead cell assay and trypan blue exclusion method^15^,^43^. Following treatment, the cells were incubated with 2 μM Calcein-AM and 4 μM EthD-1 (L3224 Invitrogen, Waltham, MA, USA) for 45 min at room temperature (RT) in dark. After washing once with phosphate buffer saline (PBS), the green fluorescence of the live cells was measured by the Gemini EX florescence plate reader (Ex/Em = 490/520; Molecular Devices, Sunnyvale, CA). In addition, trypan blue (15250, Gibco, Waltham, MS, USA) exclusion method was conducted and mean viable cell counts were calculated in 16 randomly selected areas (1 mm^2^, n = 10) to reveal the cell viability. Briefly, within 5 min after adding trypan blue, we digitally captured under microscope (200x) 10 pictures (approximately 100 cells/picture) for each condition, then randomly selected 5 pictures, and counted the number of cells for each individual treatment condition. Normalized cell viability was calculated from the following equation: viable cells (%) = [1.00 – (Number of blue cells /Number of total cells)] × 100. To precisely calibrate the cell viability, the values were standardized from fluorescence intensity and trypan blue data.

### Measurement of mitochondrial activity

Following OGD/R, reduction of 3-(4,5-dimethyl-2-thiazoyl)-2,5-diphenyltetrazolium bromide (MTT; 11465007001, Roche, Basel, Switzerland) by mitochondrial dehydrogenases was used as a measure of mitochondrial activity as previously described^43^. The optical density of solubilized purple formazan was measured at 570 nm on a Synergy HT plate reader (Bio-Tex, Winooski, VT, USA).

### Measurement of extracellular high mobility group box1 (HMGB1) levels and glutathione disulfide (GSSH) activity

After OGD/R, culture medium was centrifuged at 3,000 g, 4 °C for 15 min, and the supernatant was processed for detection of HMGB1 using an ELISA kit (amin416082, Antibody, Atlanta, GA, USA) with absorbance measured at 450 nm on a Synergy HT plate reader (Bio-Tex). Cells were treated with oxidized glutathione lysis reagent (V6611, Promega, Fitchburg, WI, USA), and GSSG activity, a biomarker of reactive oxygen species (ROS) production, was measured by luciferase activity on Spectra Max Gemini EM plate reader (Molecular Devices, Sunnyvale, CA, USA).

### Measurement of cell growth, glucose 6-phosphate dehydrogenase (G6PD)-, and glyceraldehyde 3-phosphate dehydrogenase (GAPDH)-activity

Following cell culture, the cleavage of the tetrazolium salt, WST-1 (4-[3(4-lodophenyl)-2-(4-nirtophenyl)-2H-5-tetrazolio]-1,3-benzene disulfonate; 05015944011, Roche) formazan was used as a measure cell growth. The optical density was measured at 450 nm on a Synergy HT plate reader (Bio-Tex). The levels of G6PDH- and GAPDH-activity were performed according to the manufacturer’s protocols for G6PD assay kit (ab102529, Abcam, Cambridge, MA, USA) and GAPDH ELISA kit (ab119627, Abcam), respectively.

### Western blot analysis

PRNCs were treated with CelLytic MT mammalian lysis reagent (C3228, Sigma-Aldrich) with protease inhibitor cocktail (I3786, Sigma-Aldrich). The lysate was centrifuged at 3,000 g, 4 °C for 15 min, and the supernatant was stored at −80 °C until analysis. Protein samples (4~35 μg/lane) were processed on 4~14% Tris-Glycine SDS-PAGE gel and then transferred onto a nitrocellulose membrane (162–0112, Bio-Rad, Hercules, CA, USA) at 30 V, 4 °C for 14 h. The nitrocellulose membranes were treated with PBS containing 0.1% Tween-20 and 3% non-fat milk (170–6404, Bio-Rad) for 45 min at RT. Membranes were then incubated with the primary antibodies, anti-oxytocin receptor rabbit antibody (1/10,000, ab181077, Abcam), anti-GABA_A_R α_1_ subunit rabbit antibody (1/3,000, ab3299, Abcam), anti-GABA_A_R α_2_ subunit rabbit antibody (1/1,000, ab72445, Abcam), anti-GABA_A_R α_3_ subunit rabbit antibody (1/1,000, ab72446, Abcam), anti-GABA_A_R α_4_ subunit rabbit antibody (1/1,000, ab4120, Abcam), anti-GABA_A_R α_5_ subunit rabbit antibody (1/3,000, ab10098, Abcam), anti-GABA_A_R α_6_ subunit goat antibody (1/1,000, ab117100, Abcam), anti-GABA_A_R β_1_ subunit rabbit antibody (1/3,000, ab154822, Abcam), anti-GABA_A_R β_2_ subunit rabbit antibody (1/30,000, ab16213, Abcam), anti-GABA_A_R β_3_ subunit rabbit antibody (1/1,000, ab4046, Abcam), anti-GABA_A_R γ_1_ subunit rabbit antibody (1/5,000, AMIN485542, Antibodies), anti-GABA_A_R γ_2_ subunit rabbit antibody (1/1,000, ab16213, Abcam), anti-GABA_A_R γ_3_ subunit rabbit antibody (1/500, ab13861, Abcam), anti-GABA_A_R δ subunit rabbit antibody (1/1,000, ab11048, Abcam), anti-GABA_A_R ε subunit rabbit antibody (1/500, ab35971, Abcam), anti-GABA_A_R π subunit rabbit antibody (1/5,000, ab26055, Abcam), anti-GABA_A_R θ subunit rabbit antibody (1/5,000, ARP5283, Antibodies), anti-bestrophin-1 (BST1) mouse antibody (1/3,000, NB300-164, Antibodies), anti-G6PD rabbit antibody (1/10,000, ab993, Abcam), and anti-GAPDH mouse antibody (1/10,000, ab8245, Abcam) at 4 °C for 14 h. After washing with PBS containing 0.1% Tween-20 (PBST), the nitrocellulose membrane was incubated with donkey anti-mouse IRDye800^®^CW secondary antibody (1/5,000, 926-32212, LI-COR, Lincoln, NE, USA), or donkey anti-rabbit IRDye800^®^CW secondary antibody (1/5,000, 926-32213, LI-COR), or donkey anti-goat IRDye800^®^CW secondary antibody (1/5,000, 926-32214, LI-COR) for 90 min at RT in dark. Immunoreactive detection using near-infrared fluorescence was performed according to the protocol of Odyssey^®^ Infrared Imaging System (LI-COR^®^).

### Immunocytochemistry analysis

PRNCs (8 × 10^4^ cell/well) were cultured in 400 μl Neural medium containing 2 mM *l*- glutamine and 2% B27 in the absence of antibiotics in poly-*l*-lysine 8-chamber (354632, BD Bioscience, Franklin Lakes, NJ, USA) for 3 days, then the cells were exposed to 1 μM oxytocin in the absence of reagents (control) for 3 days and fixed in 4% paraformaldehyde^15^. The cells were washed 5 times for 10 min in PBST. Then they were blocked by 5% normal goat serum (50062Z, Invitrogen, Carisbad, CA, USA) in PBST for 1 h at RT. Primary antibodies included anti-GABA_A_R α_4_ subunit mouse antibody (1/100, SMC-489, StressMarq Bioscience Inc., BC, Canada), anti-GABA_A_R β_3_ subunit mouse antibody (1/500, ab98968, Abcam), anti-GABA_A_R γ_2_ subunit mouse antibody (1/250, MABN263, Millipore, Billerica, MA, USA), anti-GABA_A_R δ subunit rabbit antibody (1/200, ab111048, Abcam), anti-GABA_A_R ε subunit rabbit antibody (1/200, ab35971, Abcam), and anti-microtubule-associated protein 2 (MAP2) chicken antibody (1/10,000, ab5392, Abcam). The cells were incubated overnight at 4 °C in primary antibody with 5% normal goat serum. The cells were washed 5 times for 10 min in PBST and then soaked in 5% normal goat serum in PBST containing corresponding secondary antibodies goat anti-mouse IgG-Alexa 405 (blue; 1/1,000, A31553, Invitrogen), goat anti-rabbit IgG-Alexa 405 (blue; 1/1,000, A31556, Invitrogen), goat anti-mouse IgG-Alexa 488 (green; 1/1,000, A11029, Invitrogen), goat anti-rabbit IgG-Alexa 488 (green; 1/1,000, A11034, Invitrogen), and goat anti-chicken IgG-Alexa 594 (red; 1/1,000, A11042, Invitrogen) for 90 min in the dark. Immunofluorescent images were visualized using confocal microscope (FV1000, Olympus, Tokyo, Japan). Control experiments were performed with the omission of the primary antibodies yielding negative results.

### Measurement of intracellular chloride ion influx

The quinolinium salt-based halide-sensitive fluorescence probe *N*-(ethoxycarbonylmethyl)-6-methoxyquinolium bromide (MQAE; ab145418, Abcam) was used as a measure of chloride ion influx activity^13^. Following cell culture, the PRNCs were incubated with 5 mM MQAE for 2 h at RT in the dark, and subsequently washed twice with NbActive 4 (BrainBit). Cells were then treated with 50 μM GABA (A2129, Sigma-Aldrich) or 10 nM GABA analog THIP, δ-GABA_A_R specific agonist^8^ (T101, Sigma-Aldrich), then fluorescence intensity was consequently measured at 0, 5, 10, and 20 min at RT. For inhibition assay, the cells were pretreated with 1 μM flumazenil (F6300, Sigma-Aldrich) or 1 μM picrotoxin (P1675, Sigma-Aldrich) or PBS (control) for 45 min at RT, and then stimulated with 50 μM GABA for 10 min at RT. Intercellular MQAE is quenched by 10 μM tributyltin chloride (T50202, Sigma-Aldrich) and 10 μM nigericin sodium salt (N7143, Sigma-Aldrich). The fluorescence intensity was measured by the Gemini EX florescence plate reader (Ex/Em = 360/460; Molecular Devices). The kinetic analysis was performed by using GraphPad Prism 6^®^ software.

### Data analysis

Data were evaluated using one-way analysis of variance (ANOVA) followed by post hoc compromised t-tests (GraphPad Prism 6^®^ software). Statistical significance was preset at *P* < 0.05. Data are represented as means ± SD from quintuplicates of each treatment condition.

## Additional Information

**How to cite this article**: Kaneko, Y. *et al*. Oxytocin modulates GABA_A_R subunits to confer neuroprotection in stroke *in vitro. Sci. Rep.* **6**, 35659; doi: 10.1038/srep35659 (2016).
